# Volar Barton Fracture and Ulnar Styloid Fracture With Distal Radial Ulnar Joint Disruption of the Right Wrist Without Neurovascular Deficit

**DOI:** 10.7759/cureus.47864

**Published:** 2023-10-28

**Authors:** Alpriya Pathan, Shivani R Uttamchandani, Medhavi V Jagzape, Pratik Phansopkar

**Affiliations:** 1 Physiotherapy, Ravi Nair Physiotherapy College, Datta Meghe Institute of Higher Education & Research (Deemed to Be University), Wardha, IND; 2 Musculoskeletal Physiotherapy, Ravi Nair Physiotherapy College, Datta Meghe Institute of Higher Education & Research (Deemed to Be University), Wardha, IND

**Keywords:** physiotherapy, external fixation, radius, internal fixation, volar barton fracture

## Abstract

Volar Barton fracture is a common condition in dislocation of the distal radius of the volar or dorsal aspect. It occurs due to violent injury or road traffic accidents. Radius is one of the most common parts to get affected. Various treatment plan is needed for this fracture like physical therapy and medication. Surgical intervention is necessary to treat the patient for a volar Barton fracture. Recovery takes time for this condition. For treatment, patient education is also important. The patient should be goal-oriented, cooperative, and motivated for the rehabilitation program. Isometric strengthening exercises for the shoulder and elbow were performed to regain the muscle's strength. The main aim is to make the patient independent of functional activities. Quality of life improved, and a rehabilitation program benefited the patient.

## Introduction

Volar Barton fractures are common and can result from high or low-energy injuries. These fractures have a bimodal distribution. In young people, it is due to high-energy trauma while in the older population, it is due to low-energy trauma. For unstable fractures, there are a variety of closure methods like plaster fixation using pins, external fixation with plating, or bone grafting, as well as other treatment options. However, with certain fractures, surgical treatment is preferred [1]. The most desirable results have been obtained with open reduction with volar buttress plating or closed reduction with external fixation and percutaneous Kirschner wire pinning. Internal fixation as well as open reduction are necessary due to the inherent instability of the functional and radiological results [2]. Because of the possibility of carpal subluxation, displaced intraarticular distal radius fractures have a worse prognosis than extraarticular fractures due to intercarpal ligament injuries, and arthrosis of the radiocarpal and distal radioulnar joints. Various treatment modalities, including closed reduction and casting, percutaneous pinning, external fixators, and open reduction and internal fixation, are being described for intraarticular distal radial fractures. The preferred method is open reduction and internal fixation because it allows reduction with direct vision, stable internal fixation, a shorter period of surgery or mobilization, rapid recovery from immobility, and returns to function. The T-plate is used in the treatment of these fractures to achieve union and good hand function. Patients with volar Barton's fractures who underwent surgery using a small buttress T-plate were examined to predict the functional outcome. The conservative approach is frequently ineffective and rife with problems like instability, subluxation, early osteoarthrosis, and deformity. However, there hasn’t been much encouraging research on how well surgical treatment works [3]. Distal radius and ulna are reinforced and stabilized by a variety of treatment approaches, ranging from closed reduction and immobilization to open reduction with plates and screws

## Case presentation

A 33-year-old male who had a road traffic accident came to the hospital, a vehicle dashed him and he fell off. After a few hours, swelling and pain occurred in his right wrist and he was not able to perform movements. The pain gradually increased so he went to a nearby village hospital some investigation was done including an X-ray. According to the investigation, the distal radius was fractured. The doctor suggested an operation in which open reduction and internal fixation with plate osteosynthesis for volar Barton fracture of the wrist was done. The fracture was fixed with a two-hole distal end radius volar plate.

Clinical findings

**Figure 1:**
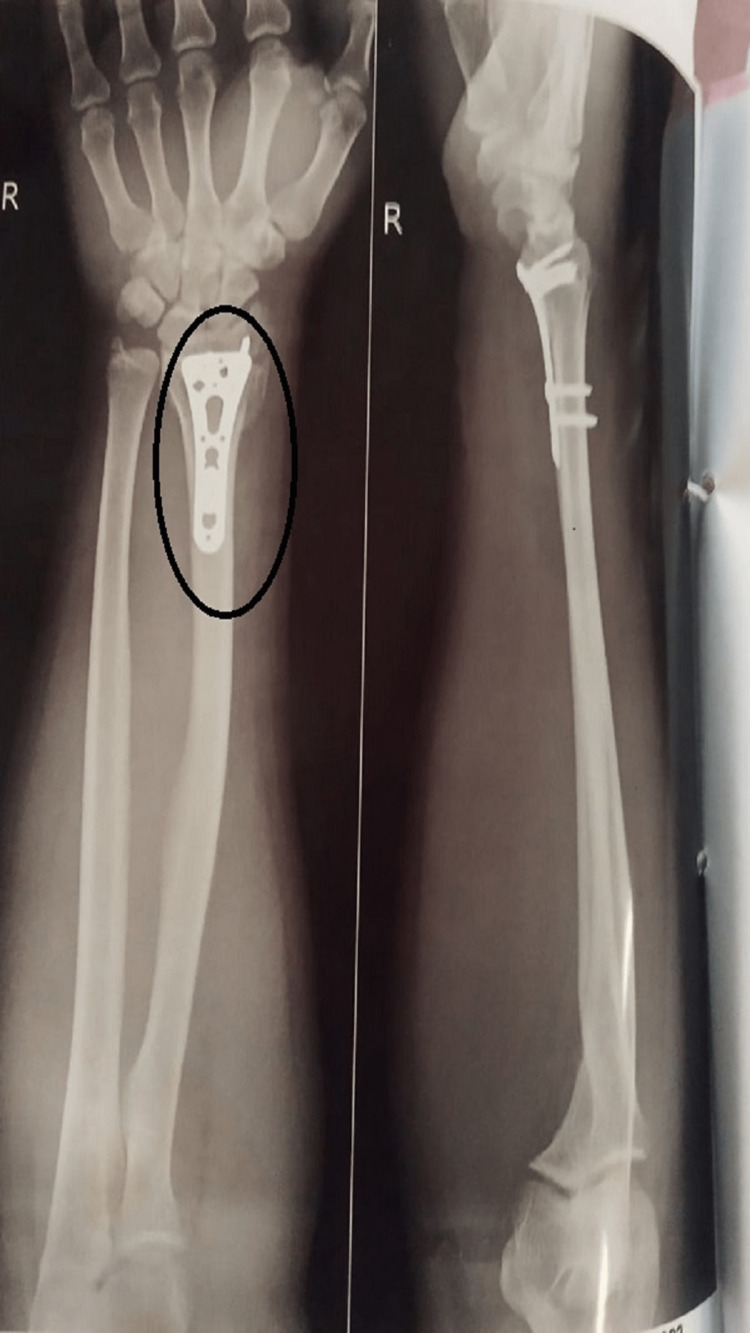
Post-operative X-ray of the wrist

On general examination, the patient was oriented to place and time, well-motivated, and cooperative. The patient was moderately built and afebrile and his vitals were stable. On observation, the limb is in a side-lying position, shoulder abducted, and forearm pronated. There is no deformity present on the hand and the gait is normal. Palpation revealed grade 1 tenderness and moist skin type and there was no spasm present. Table 1 shows the range of motion, Table 2 shows manual muscle testing, and Table 3 shows the numerical pain rating scale upon examination. The postoperative X-ray is seen in Figure 1. After one day of surgery, these post-operative X-rays were taken.

**Table 1:** Range of motion of the right-hand affected extremity

	Passive	Active
Wrist flexion	25°	10°
Wrist extension	30°	15°
Elbow flexion	70°	50°
Elbow extension	80°	60°
Pronation	50°	20°
Supination	20°	15°

**Table 2:** Manual muscle testing of the right-hand affected extremity

	Grades
Elbow flexors	3/5
Elbow extensor	3/5
Pronators	3/5
Supinators	3/5

**Table 3:** Numerical pain rating scale

Numerical pain rating scale	7/10

Intervention

Therapeutic intervention is shown in Table 4.

**Table 4:** Weekwise intervention

Weeks	Interventions	Regimen
Week 1	To reduce pain and edema cryotherapy was applied. The elbow's range of motion is active and actively assisted, and the forearm's thumb and finger movements are passive. Isometric exercises to abductor digiti minimi were performed by the patient while keeping the forearm in a mid-prone position, the thumb flexors and extensors, as well as the finger flexors and extensors, were stretched.	For 10 minutes, 10 repetitions, two times a day were given. 10 repetitions, two times a five-day repetition, two times a day
Week 2-4	Full active range of motion of metacarpal and interphalangeal joint performed by the patient. Squeezing a sponge ball helps the patient improve grip strength. Scapular sets performed by the patient.	10 repetitions, two times a day, 10 repetitions, two times a day, 10 repetitions, two times a day
Week 5-8	As swelling subsided cryotherapy was discontinued. Isometric strengthening exercises were initiated for the shoulder and elbow. The therapist provided resistance while encouraging the patient to initiate flexion, extension, supination, and pronation.	10-sec hold with 10 repetitions
Week 8-10	Elbow flexors were statically stretched, and an active range of motion was performed. Full range of motion active and passive in all planes performed by the patient. Exercises focused on performing functional tasks were started to ease the difficulty of daily tasks. Self-stretching was taught to the patient.	10 repetitions, 2 times a day. 10 repetitions, 2 times a day. 10 repetitions, 2 times a day

Outcome measures

The range of motion of the affected extremity is shown in Table 5, manual muscle testing is shown in Table 6, and the numerical pain rating scale is shown in Table 7.

**Table 5:** Range of motion of the affected extremity

	Passive	Active
Wrist flexion	30°	15°
Wrist extension	34°	20°
Elbow flexion	74°	55°
Elbow extension	84°	65°
Pronation	54°	25°
Supination	25°	20°

**Table 6:** Manual muscle testing of the affected extremity

	Grades
Wrist flexors	4/5
Wrist extensors	4/5
Pronators	4/5
Supinators	4/5

**Table 7:** Numerical pain rating scale

Numerical pain rating scale	4/10 on movement

## Discussion

Minimal gap, proper stability, and adequate nutrient supply are factors that promote fracture healing [5]. Wrist and finger range of motion exercises should be started as soon as possible to prevent poor joint function. This is crucial for Barton's fractures with intraarticular involvement. Once there is joint adhesion, treatment is very difficult. Both intra and extraarticular release techniques are very challenging. Joint immobilization in these instances lasted no longer than six weeks. The restricted movement of the thumb and index finger is the cause of the less-than-satisfactory cases. Some patients are still unable to effectively flex their fingers six weeks after removal of the Kirschner wires and external fixation. External fixation pins must be carefully positioned as a result. Then, the success rate will increase. A volar's malunion Barton's fracture is very challenging to treat and can cause severe disability. The articular cartilage could also be seriously injured and unable to recover. Therefore, it is crucial to prevent a malunion in a volar Barton's fracture in order to avoid having to treat malunion. Kirschner pinning in conjunction with plating or external fixation provided enough stability for the fracture to heal. More precisely reducing and stabilizing fragments are possible with plating treatment. But doing so necessitates opening the fracture site. Closing the incision site may be very challenging, if there is significant localized swelling, though typically, the incision is not very large, and the infection rate is negligible. Moreover, the fracture healing procedure is unaffected by the cancellous bone's characteristics. With plating treatment, pieces can be reduced more precisely and stabilized more firmly. Opening the fracture site is necessary though. The cancellous bone’s characteristics also prevent the healing of fractures from being hampered. Therefore, there is a high success rate. The ulnar styloid serves as a strut, aiding in the stabilization of the ulnocarpal ligaments, the extensor carpi ulnaris, and its subsheath [6]. A common complication of volar Barton's fractures, an unstable distal radial fracture, is subluxation or luxation of the wrist joint. The main objectives of treatment for this injury are to provide an excellent reduction and quick stabilization in order to achieve anatomic fracture union, expedite the recovery of hand function, and prevent sequelae. Fracture healing needs a small gap, the right stability, and enough blood flow. As a result, the locking plate is preferred for fracture healing. Grip strength, lateral pinch strength, and wrist range of motion for the affected side are noted. The average wrist flexion, loss in pinch, and grip strength were all 86% of the normal side. The system uses clinical examinations such as grip strength measurements, radiography, and patient-related wrist evaluation ratings. Closed techniques are frequently ineffective for stabilizing unstable intraarticular fractures and restoring the wrist interarticular unstable intraarticular fractures and the wrist's interarticular integrity and radial length; therefore, various surgical methods are used [7]. The goal of treating unstable fractures of the distal radius is to restore the damaged anatomy as best as possible, enable rapid restoration of hand function, and prevent secondary fracture displacement [8]. It has been demonstrated that early wrist motion improves hand and finger function. Advances have been sought with the help of modern improvements in techniques for internal and external fixation.

## Conclusions

Physiotherapy aids in enhancing physical capacity and independent living. The patient was given a thorough recovery plan that assisted him in reducing pain and edema and increasing his range of motion. The patient was able to carry out daily activities after completing his or her regular exercises. The patient is well cooperative and motivated towards the treatment protocol. Carelessness and a lack of patient awareness could lead to more complications. Prevention measures were taken while treating the patient.
